# The role of nurses in psychoeducation for improving awareness the dangers of bullying among students: a quasi-experimental study

**DOI:** 10.1186/s12912-025-03991-0

**Published:** 2025-12-05

**Authors:** Iyus Yosep, Rohman Hikmat, Ai Mardhiyah, Heni Purnama

**Affiliations:** 1 Department of Mental Health, Faculty of Nursing, Universitas Padjadjaran, Sumedang, Jawa Barat Indonesia; 2 Doctoral Students of Nursing, Faculty of Nursing, Prince of Songkla University, Hat Yai District, Songkhla, 90110 Thailand; 3 Department of Pediatric Nursing, Faculty of Nursing, Universitas Padjadjaran, Jawa Barat, Sumedang, Indonesia; 4 Department of Mental Health, STIKEP PPNI Jabar, Jawa Barat, Indonesia

**Keywords:** Bullying, Nurses, Psychoeducation, Students

## Abstract

**Introduction:**

Bullying is a persistent, aggressive behavior characterized by a significant power imbalance that severely affects students’ mental and social well-being. Moreover, nurses serve as health educators in schools. They are well positioned to deliver psychoeducation that raises awareness of bullying and supports prevention.

**Objective:**

To determine the effect of nurse-led psychoeducation on high school students’ awareness of the dangers of bullying in Bandung, Indonesia.

**Method:**

A quasi-experimental, pre- and post-test design was employed with a control group and an intervention group. A total of 100 high school students in Bandung were randomly assigned, with 50 in the intervention group and 50 in the control group. For three months, nurses delivered four psychoeducation sessions on bullying to the intervention group. Meanwhile, the control group continued routine activities. Awareness was measured using the Bullying Danger Awareness Questionnaire before and after the intervention. Data analysis was performed using the Wilcoxon and Mann-Whitney tests.

**Results:**

The intervention group showed significant improvement in awareness of the dangers of bullying compared to the control group (*p* < 0.001).

**Conclusion:**

Nurse-led psychoeducation improves students’ awareness of bullying risks. Future studies should evaluate behavioral change and longer-term effects using larger samples and follow up.

**Clinical trial number:**

Not applicable.

## Introduction

Bullying is aggressive behavior that is carried out repeatedly with the intention of hurting or oppressing another individual. Bullying involves a power imbalance in which perpetrators hold physical, psychological, or social advantages over victims [1]. This imbalance can manifest in the form of physical strength, social popularity, or access to information that can be detrimental to the victim. Bullying takes physical, verbal, and psychological forms, for example: hitting or kicking; insults or teasing; and spreading rumors or social exclusion [2]. Because of its repetitive nature, bullying tends to have long-term detrimental effects, not only for the victim but also for the perpetrator and bystanders [3].

The prevalence of bullying among high school students constitutes a significant global problem. Based on research conducted in 83 countries, findings revealed that the global prevalence of bullying reached 30.5% among students aged 12 to 17 years [4]. In the United States, about 20% of high school students report being bullied; social exclusion and teasing are the most common, cited by roughly 70% of students [5]. In Egypt, bullying prevalence among high school students reached 77.8%, showing that bullying occurs across diverse social contexts, not only in developed countries [6]. Research in Taiwan also showed that cyberbullying has emerged as an increasingly prevalent phenomenon, with incidence rates reaching 35.4% among students [7].

In Indonesia, the prevalence of bullying among high school students represents a pressing concern. According to research conducted by Shahrour et al., (2020), the prevalence of bullying in Indonesia reached 27.7%, with the most common forms of bullying being verbal and physical [2]. The study also showed that peer support and the school environment strongly influence bullying levels among students [8]. The Global School-based Student Health Survey found that bullying in Indonesia harms students’ mental health. Many students reported stress and anxiety linked to bullying [9].

Bullying has short and long-term effects on victims, perpetrators, and their social environments. In the short term, victims often develop anxiety, depression, and low self-esteem, which hinder social and academic functioning [10, 11]. Research demonstrates that victims of bullying tend to exhibit lower academic achievement and increased absenteeism from school, which contributes to educational quality deterioration [12]. On the other hand, perpetrators also face negative consequences, including heightened propensity for aggressive behavior and social maladjustment later in life [13]. Over time, both victims and perpetrators may develop severe mental health problems, such as PTSD, and face a higher risk of criminal behavior [14]. Previous studies show that those involved in bullying (victims or perpetrators) are more likely to have adult mental health problems, including persistent depression and anxiety [15].

A shortage of programs that build students’ awareness of bullying risks contributes to its high prevalence in schools. Some schools have anti-bullying policies, but many lack ongoing, structured education about harms to both victims and perpetrators [10]. Previous studies show many students do not recognize the psychological consequences for victims such as depression and anxiety, or the long-term effects on social and academic paths [16]. Perpetrators are often unaware that their actions can lead to future aggressive behavior and serious interpersonal difficulties [17].

Psychoeducation is an educational intervention that improves knowledge and understanding of psychosocial issues including bullying. Psychoeducation aims to provide comprehensive information about the various manifestations of bullying behavior, its negative impacts, and effective prevention strategies [18]. Psychoeducation can explain consequences for victims and perpetrators and motivate students to help create a safe, supportive school environment [19, 20]. Structured, interactive psychoeducation raises awareness of mental health and social behavior, helping students better recognize and respond to bullying [21].

Many studies examine psychosocial interventions to curb adolescent bullying, but few assess psychoeducation’s effect on students’ awareness, especially in Indonesia [22–24]. Most studies focus on the behavior or psychology of victims and perpetrators, not on how systematic education builds cognitive awareness about bullying [25–27]. Elevating awareness constitutes an essential first step in fostering preventive and empathetic attitudes toward bullying in educational environments. However, most of these studies have only measured awareness or knowledge, without examining more comprehensively how the intervention affected bullying behavior itself. More research should test how increased awareness relates to behavior, since awareness alone may not prevent bullying. Therefore, this study aims to address this gap by investigating the impact of psychoeducational interventions on anti-bullying awareness among high school students in Indonesia. The purpose of this study was to determine the effect of nurse-led psychoeducation on high school students’ awareness of the dangers of bullying in Bandung, Indonesia.

## Materials and methods

### Study design

This study used a quasi-experimental pretest and posttest design to compare awareness before and after psychoeducation. The intervention group received four sessions across three months, while the control group continued regular activities. Groups were randomized by list, but students stayed in their classes, creating unadjusted cluster effects.

### Participant

The study population consisted of 800 high school students in Bandung. Power analysis with G Power indicated at least 74 participants to detect group differences with a large effect and a power of 0.95. Inclusion criteria were age 15 to 18, consent to join, and no bullying involvement in the past year per school records. Students with physical or psychological conditions that could hinder participation were excluded. Participants were chosen by proportionate stratified sampling by grade, then randomized to intervention and control by an online generator. The final sample of 100 exceeded the target and yielded the power of about 0.95.

### Procedure

- Pre-Experiment Measurement: Before the intervention, all participants completed the Bullying Danger Awareness questionnaire for baseline awareness. The results were used to screen for inclusion eligibility and to serve as the baseline measurement. Eligible students were then randomly assigned to the intervention and control groups.
- Implementation of Psychoeducation: The intervention group attended four bullying psychoeducation sessions over three months. Each session lasted 60 min. To minimize contamination, each group was allocated to a different class section. No matching or stratification by baseline awareness was used, which may have introduced imbalance. The following are the details of the psychoeducation sessions provided:

**Session 1: Definition and Types of Bullying.** This session focused on introducing bullying, including the definition, its forms (physical, verbal, and social), and how bullying occurs in various school contexts.

**Session 2: The Impact of Bullying on Victims.** In this session, students were given information about the psychological, social, and academic impacts experienced by victims of bullying. This material was accompanied by case studies and testimonies from victims of bullying.

**Session 3: How to Prevent Bullying.** Students were taught strategies to prevent bullying, both from an individual perspective, such as having the courage to report, and from a group perspective, such as creating an environment that supports anti-bullying initiatives.

**Session 4: Supporting Victims of Bullying and Being Proactive.** This final session emphasized the importance of supporting victims of bullying and how students can be proactive in preventing bullying through empathy and collaboration.

c) Post-Experimental Measurement: After the sessions both groups repeated the questionnaire to assess change in bullying awareness. The intervention group also attended a closing session involving a reflective discussion to strengthen their understanding and commitment to bullying prevention.

### Data collection

We used the Bullying Danger Awareness Questionnaire with 24 items to assess cognitive awareness and emotional attitudes about bullying in school settings [28]. Each item used a four-point Likert scale such as Strongly Agree, Agree, Disagree, and Strongly Disagree, with reverse scoring for negatively worded items. The questionnaire includes both positively and negatively framed statements to minimize response bias. The instrument measured three constructs awareness of forms and consequences, empathy for victims, and readiness to act. Three experts in nursing and adolescent psychology evaluated content validity.

Item total correlations were 0.452 to 0.813 and Cronbach’s alpha was 0.834 (valid and reliable). Data were collected at two points: pre-intervention and post-intervention in both groups. For analysis, the total scores were categorized into three groups: low (50 or less), moderate (51 to 70), and high (71 or more). This classification was based on percentile distribution and expert agreement during instrument validation.

### Data analysis

Data were analyzed using univariate and bivariate tests. Univariate analysis described demographics (gender, age, grade) with frequencies, percentages, means, minima, maxima, and standard deviations. Because scores were non-normal by Kolmogorov-Smirnov test, we used Wilcoxon test for within-group change and Mann-Whitney U test for between-group comparisons. We reported p-values and effect sizes r using the standard Z over root N formula to show practical significance. Analyses were performed in SPSS version 24. ANCOVA or regression could adjust baseline differences but was not feasible due to the small sample and non-normality, and future studies should use these with larger samples.

### Ethical considerations

Ethical approval was granted by the Research Ethics Commission of STIKEP PPNI West Java Province No. III/020/KEPK-SLE/STIKEP/PPNI/JABAR/VI/2022. This study followed the Declaration of Helsinki, obtained written consent from students and guardians, and ensured voluntary participation with a right to withdraw without consequence. Anonymity and confidentiality were protected with coded identities, secure password-protected storage, restricted access, and locked files for print records. Veracity was upheld by clearly explaining objectives, procedures, benefits, and minimal risks. Beneficence guided the design to raise awareness, nonmaleficence was ensured with age- appropriate content and safe settings, and justice was maintained with equal participation across gender, class level, and performance.

## Results

**Table 1:** Characteristics of respondents (*n* = 100)

Demographic Variables	Total	Percentage
**Gender**		
Male	46	46%
Female	54	54%
**Age**		
15 years	11	11%
16 years	37	37%
17 years	46	46%
18 years	6	6%
**Class**		
X	23	23%
XI	55	55%
XII	22	22%

Based on Table 1, out of 100 respondents, the majority were female, comprising 54%, while the remaining 46% were male. The age distribution shows that 46% of respondents were 17 years old, followed by 37% who were 16 years old. The age group of 15 years old accounted for 11%, and 6% of respondents were 18 years old. In terms of grade, the majority of respondents were in grade XI, with 55% of them. Grade X followed with 23%, and grade XII had 22%.

**Table 2:** Descriptive analysis test results (*n* = 100)

Group	Minimum	Maximum	Mean	Std. Deviation
Pre-test Control	40	88	60.32	11.15
Post-test Control	42	90	61.28	10.63
Pre-test Intervention	38	85	58.96	11.92
Post-test Intervention	56	96	71.48	10.23

**Fig. 1:**
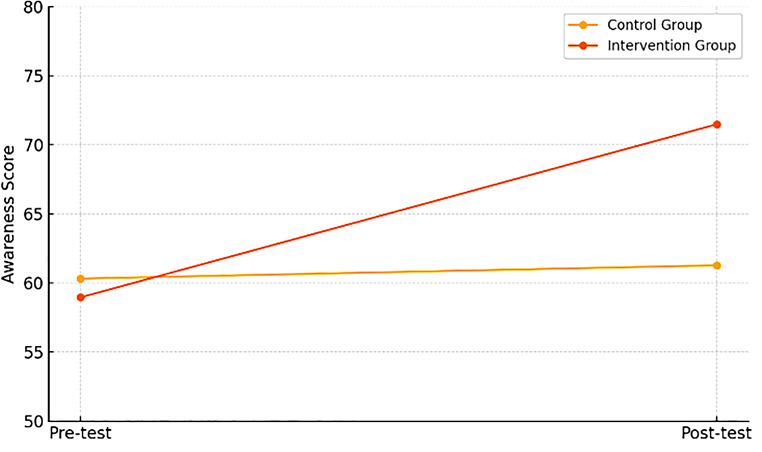
Increasing awareness of the dangers of bullying

Based on Table 2, in the control group, awareness scores showed only a slight increase from pre-test (M = 60.32) to post-test (M = 61.28). In contrast, the intervention group demonstrated a marked improvement, rising from pre-test (M = 58.96) to post-test (M = 71.48). This indicates that the psychoeducational sessions were effective in significantly increasing awareness of the dangers of bullying, while changes in the control group were minimal (Fig. 1).

**Table 3:** Frequency distribution of levels of awareness of the dangers of bullying in the experimental group (*n* = 50)

Bullying Danger Awareness	Pre-Intervention		Post Intervention	
	∑	%	∑	%
Low	14	28%	3	6%
Medium	22	44%	17	34%
High	14	28%	30	60%

Based on Table 3, before the intervention, most respondents in the experimental group showed moderate awareness (22 participants, 44%). The remaining participants were equally divided between low and high awareness, with 14 participants each (28%). After the psychoeducational sessions, the distribution shifted. High awareness increased to 30 participants (60%). Moderate awareness decreased to 17 participants (34%), and only 3 participants (6%) remained in the low category.

**Table 4:** Frequency distribution of levels of awareness of the dangers of bullying in the control group (*n* = 50)

Awareness of the dangers of bullying	Pre-Control		Post Control	
	∑	%	∑	%
Low	13	26%	10	20%
Medium	25	50%	27	54%
High	12	24%	13	26%

Based on Table 4, in the pre-test, most respondents in the control group were in the moderate category (25 participants, 50%). This was followed by 13 participants (26%) in the low category and 12 participants (24%) in the high category. After the observation period, the distribution changed slightly. The moderate group increased to 27 participants (54%), the high group rose to 13 participants (26%), and the low group decreased to 10 participants (20%).

**Table 5:** Wilcoxon test of the effect of psychoeducation on the level of anti-bullying danger

Value components	Post-intervention - Pre-intervention
Z	-5.012
Asymp. Sig. (2-tailed)	< 0.001
Mean difference	25.00
Effect size	0.709

Based on Table 5, the Wilcoxon signed rank test showed a significant increase in the intervention group (*p* < 0.001). The effect size was *r* = 0.709 which shows strong practical impact. The test yielded Z = -5.012 and a mean rank difference of 25.00.

**Table 6:** Mann-Whitney test of the differences in anti-bullying danger post-test scores in the control and intervention groups

Measurement	Results
Mann-Whitney U	602.000
Z	-4.126
Asymp. Sig. (2-tailed)	0.000
Effect size	0.413

Based on Table 6, the Mann-Whitney U test showed a significant difference between groups (*p* < 0.001). U = 602.000 and Z = -4.126. The effect size was *r* = 0.413 which indicates a moderate to large effect. These findings show that the intervention meaningfully improved awareness compared with the control group.

## Discussion

This study aims to evaluate the effectiveness of psychoeducational interventions in enhancing awareness of the dangers of bullying among high school students. Bullying, as a detrimental social phenomenon, often occurs in educational environments and has negative impacts on the mental health, social well-being, and academic achievement of students [29]. Psychoeducational interventions help students understand the negative impacts of bullying and motivate them to prevent it [30].

The involvement of nurses in psychoeducational interventions significantly enhances students’ awareness of the dangers of bullying. Acting as both information providers and facilitators, nurses guide students through reflection, discussions, and value-building while using their communication and empathy skills to create a supportive environment where students feel safe sharing their opinions and experiences [23, 31]. By leading sessions that connect cognitive and emotional learning, nurses ensure that anti-bullying messages are understood and applied in daily life [32]. These findings align with previous studies showing that nurses improve comprehension, attitudes, and behavior change [21]. This study adds further evidence by positioning nurses as primary implementers rather than auxiliary support, highlighting their professional capacity in school-based health education [33]. Nurse led programs integrate education with psychosocial support more effectively than teacher or counselor programs, making their inclusion essential for best outcomes. Institutional policies should support nurse involvement in school psychoeducation to build a safe and violence free learning environment [34, 35].

Psychoeducational interventions can effectively enhance students’ awareness of bullying, particularly in collectivistic societies like Indonesia. The improvement in awareness scores shows that structured and empathetic sessions effectively build both understanding and emotional engagement [36]. This aligns with evidence that psychosocially based education more effectively shapes adolescent attitudes and behavioral intentions [37]. Nurse facilitation highlights the public health education partnership and underscores nurses strategic roles in school mental health promotion and violence prevention [25].

The increase in awareness scores (58.96 to 71.48) shows the strong impact of the intervention. This large effect aligns with reviews showing that school psychoeducation and anti-bullying programs yield small to moderate gains in awareness and reduce bullying [21, 29, 38–40]. Our study shows a larger effect, likely reflecting the structured role of nurses as facilitators. In Indonesia, strong respect for authority figures like teachers and nurses may have amplified receptiveness to nurse-delivered psychoeducation [41, 42]. These results suggest effectiveness depends on both content and sociocultural context.

The difference in outcomes between the intervention and control groups confirms the effectiveness of the focused intervention program, as shown by higher post-test scores and a moderate-to-large effect size (*r* = 0.413). Previous studies have also found that interventions specifically designed to enhance awareness of bullying are more effective than non-focused or routine programs [23, 43]. Well-structured psychoeducational programs that combine information delivery with group discussions improve understanding, empathy, and attitudes, leading to positive behavioral changes [44–46]. Psychoeducational interventions using information delivery and group discussions can increase bullying awareness and reduce its occurrence [45–47]. These findings suggest that focused interventions not only increase awareness but also promote nuanced reflection on bullying, emphasizing the importance of deliberate program design for optimal outcomes.

In Indonesia, bullying is often normalized and not recognized as psychosocial violence [48]. Phrases like “just joking” to get closer or something similar in schools are used to excuse belittling mocking or intimidating peers. This normalization leads many students teachers and parents to overlook the serious psychological harm [37]. This shared unawareness underscores the need for education that builds awareness of bullying definitions forms and consequences. Psychoeducation can shift perceptions so bullying is no longer seen as a normal part of school life [49]. Raising awareness is a key prerequisite for a safe inclusive and violence free school culture.

These findings suggest the importance of integrating continuous psychoeducational programs into school curricula [18, 40, 50, 51]. Such programs should teach the effects of bullying, foster empathy, and involve students, teachers, and parents. Nurses also play a key role by recognizing bullying, providing empathy-based interventions, and leading awareness sessions in schools or communities [23, 52, 53]. Their role includes facilitating educational sessions in clinics or community health units to enhance awareness of the dangers of bullying and ways to prevent it. Embedding these interventions within Indonesia’s cultural context enhances their impact, as the authoritative role of nurses and the country’s collectivist values support acceptance of anti-bullying messages.

### Limitations

This study has several limitations. First, it measured awareness of bullying rather than actual behavior change, so results reflect cognitive understanding only. Second, the small sample size (*n* = 100) and possible baseline differences limit generalizability, highlighting the need for better matching or covariate adjustments. Third, while the Bullying Danger Awareness Questionnaire showed acceptable validity and reliability, further psychometric testing is needed. Fourth, the quasi-experimental design without randomization may introduce selection bias. Fifth, the short intervention period and lack of follow-up prevent evaluation of long-term effects. Future studies should assess sustained awareness and behavioral outcomes, such as reduced bullying or improved bystander responses, to better understand intervention impact.

## Conclusion

This study showed that psychoeducation significantly increased students’ awareness of bullying, as reflected by score improvements. However, since it measured awareness rather than behavior, it remains unclear whether these gains lead to actual behavior change. Future research should include behavioral outcomes to determine if psychoeducation reduces bullying and improves school climate.

Nurses have a role in delivering psychoeducation as part of school-based health promotion. The results support integrating such programs into schools to strengthen students’ understanding of bullying. Future studies should also explore scalable approaches, such as digital or telehealth delivery, for broader and more sustained impact. In practice, nurse-led programs can be implemented through workshops, classroom sessions, or inclusion in health curricula, supported by collaboration with teachers and counseling staff.

## Data Availability

The data that support the findings of this study are available from the corresponding author, [IY], upon reasonable request.
